# American Indian and Alaskan Native Access to Obstetrics and Gynecology Subspecialists: Findings From a National Mystery Caller Study in the United States

**DOI:** 10.7759/cureus.51403

**Published:** 2023-12-31

**Authors:** Adeola O Akapo, Claire Schultz, Diego Coelho, Tyler M Muffly

**Affiliations:** 1 Obstetrics and Gynecology, University of Colorado Anschutz Medical Campus, Aurora, USA; 2 Obstetrics and Gynecology, Denver Health and Hospital Authority, Denver, USA; 3 Biostatistics and Epidemiology, Coelho Services, Florianópolis, BRA

**Keywords:** subspecialty care, indian reservation, obstetrics and gynecology services, obstetrics and gynecology, geographical analysis, indian health service, alaska native, american indian

## Abstract

Background

A significant disparity exists for American Indian and Alaska Native populations in accessing obstetric and gynecology (OBGYN) subspecialty care, as nearly 43% of individuals do not reside in areas where the Indian Health Service (IHS) provides care. Geographical separation from IHS facilities exacerbates healthcare disparities, particularly regarding access to specialized services. This study aims to create a map illustrating the average driving time from an IHS clinic to OBGYN subspecialists (e.g., gynecologic oncology, maternal-fetal medicine, family planning, urogynecology, pediatric and adolescent gynecology, and reproductive endocrinology and infertility [REI]) and determine the average wait time for appointments with these specialists.

Study design

A cross-sectional and mystery caller study was conducted using hospital-level data from the IHS and data on women from the 2010 United States Census provided by the US Census Bureau. All US OBGYN subspecialists were identified and mapped. The local distribution of clinics near IHS hospitals was determined, and the nearest OBGYN subspecialist was mapped to IHS hospitals providing women's care services. Thirty-seven OBGYN subspecialists closest to IHS hospitals were contacted to calculate the mean wait time for subspecialty care appointments.

Results

The median driving time to the closest gynecologic oncology, maternal-fetal medicine, family planning, urogynecology, pediatric and adolescent gynecology, and reproductive endocrinology and infertility OBGYN subspecialist was 214 minutes (interquartile range [IQR] 107-290). The longest drive to see a subspecialist for urogynecology services was over 240 minutes. From the 2010 US Census, we identified 583,574 American Indian and Alaska Native (AI/AN) pediatric, adolescent, and women within a 60-minute drive of an IHS hospital. The mean wait time for a new patient appointment was 13.6 business days (SD ± 2).

Conclusions

Geographical disparities significantly impact the ability of American Indian and Alaska Native populations to access OBGYN subspecialty care. There was no difference in wait times compared to the national average, though there were significantly longer drive times.

## Introduction

The partnership between the American Congress of Obstetrics and Gynecology and the Indian Health Services (IHS) aims to address healthcare barriers faced by American Indians and Alaska Natives (AI/AN) [1]. The IHS is a government agency responsible for providing healthcare services to approximately 2.2 million enrolled members of federally recognized tribes, primarily through the IHS and tribal hospitals and clinics. The combined size of AI/AN reservations would be larger than the state of Michigan, both institutions with billion-dollar budgets. The IHS provides and funds healthcare services for eligible AI/AN who receive healthcare through various facilities near reservations, including the IHS and tribal hospitals and clinics. These disparities extend to areas such as premature birth rates and late-stage breast and cervical cancer diagnoses, which are significantly higher among AI/AN populations than white populations [2]. Specifically, rates of inadequate prenatal care and postneonatal death among AI/AN infants are two to three times those of white infants and even higher among rural AI/AN infants [3-4].

The healthcare disparity is even more significant when access to subspecialty care is considered. Wait time is a quantitative metric of access to healthcare. Eligible AI/AN individuals can receive healthcare at any IHS facility. Still, complex rules govern and restrict the delivery of specialty medical care, which is unavailable at IHS facilities. When patients require the care of an obstetrics or gynecology (OBGYN) subspecialist, their primary OBGYN provider will refer them to a provider outside the IHS system. Specialty care can be obtained by patients through purchased or directed care (PRC), where health services are provided at the expense of the IHS from public or private sector medical facilities [5]. This system allows AI/AN individuals to access specialty care that is unavailable through the IHS. Telehealth OBGYN subspecialty care is not widely available, and OBGYNs use telehealth (9.3% of visits), which is half that of other surgical specialties [6-9].

This cross-sectional and mystery caller study aimed to assess the geographic distribution of practicing OBGYN subspecialists in the following subspecialties: gynecologic oncology, maternal-fetal medicine, family planning, urogynecology, pediatric and adolescent gynecology, and reproductive endocrinology and infertility (REI) in the United States near AI/AN IHS hospitals and determine the travel distance required to reach these subspecialists from IHS facilities. Additionally, the study aimed to investigate the wait time for new patient visits to OBGYN subspecialists through a mystery caller study. This is the first study to examine such factors concerning OBGYN subspecialty care in this population.

## Materials and methods

This study employed a geographic analysis and a mystery caller-style telephone survey to assess the accessibility of obstetrics and gynecology subspecialty care for AI/AN patients at IHS facilities (Table 1). The study was deemed exempt from review by the Colorado Multiple Institutional Review Board.

**Table 1:** Scripted clinical vignettes used during mystery calls.

Specialty type	Medical condition	Age	Referral source	Symptoms
Urogynecology	Stress urinary incontinence	65 yr	Primary care provider	Leaking when runs and coughs started five years ago, PCP has tried pelvic floor physical therapy
Gynecologic oncology	New pelvic mass	65 yr	Primary care provider/gynecologist	Early satiety, pelvic pressure, and the emergency department noted unilateral fixed 10 cm mass
Maternal-fetal medicine	Preconceptual counseling autologous kidney transplant	35 yr	Primary care provider	A nulliparous patient who received an autologous kidney transplant desiring preconceptual counseling
Reproductive endocrinology and infertility	Primary infertility	35 yr	Primary care provider	Desires to have a child but is unable to conceive after one year of unprotected sex with a partner who has fathered children previously
Pediatric and adolescent gynecology	Anovulatory bleeding	13 yr	Primary care provider	The caller is the 13 year old patient’s parent; irregular menstrual bleeding

We used the term IHS as shorthand for federally funded healthcare facilities operated by the IHS; this terminology does not include tribal healthcare facilities funded through other mechanisms. We used American Indian, rather than Native American, to describe indigenous people of the continental US, given this term’s use in the US Census Bureau. We also recognized its fraught history of terminology and the variation in individual preferences. We assessed timely access to obstetrics and gynecology subspecialty care using three approaches. First, we estimated driving times and distances to the closest OBGYN subspecialist from the 28 AI/AN hospitals providing OBGYN services in the US. Second, we utilized driving times to ascertain the percentage of individuals, specifically pediatrics, adolescents, and women, who could access an OBGYN subspecialist within a 30-minute timeframe [10]. To provide a more comprehensive understanding of this aspect of the study, it is recommended to incorporate additional information regarding the age groups considered and any other relevant criteria employed during the search process. Third, we performed a mystery caller study on the subspecialists closest to the IHS hospitals to determine wait times for patients with IHS insurance.

Data sources

The primary data sources for this study were the US Census Bureau and the National Plan and Provider Enumeration System [11]. The US Census Bureau provided information on the number of female AI/AN individuals and their geographic locations at the block group level [12]. The US Census Bureau typically collects and provides demographic data based on age groups such as 0-17 (pediatric), 18-24 (adolescent/young adult), and older than 24 for women. The IHS Chief Clinical Consultant for OBGYN identified the local addresses of the 17 IHS hospitals providing inpatient obstetrics and gynecology services. Geocoding was performed using R code to map the addresses [13]. Subspecialty obstetrician-gynecologists (e.g., gynecologic oncology, maternal-fetal medicine, family planning, urogynecology, pediatric and adolescent gynecology, and reproductive endocrinology and infertility) were identified using taxonomy numbers from the National Plan and Provider Enumeration System. Although the National Plan and Provider Enumeration System represents one of the largest and most comprehensive registries of US physicians, it includes voluntary and self-reported data on select attributes. Inclusion criteria included the subspecialty physicians (gynecologic oncology, maternal-fetal medicine, pediatric and adolescent gynecology, reproductive endocrinology and infertility, and urogynecology) who had an address located nearest an IHS hospital. Specifically, we selected board-certified. This criterion ensured that the selected subspecialty physicians were readily accessible to patients seeking specialized obstetrics and gynecology services within the IHS network.

Geographic analysis

To assess the geographic accessibility of OBGYN subspecialists within the IHS system, the subspecialty practice locations were plotted on a map that displayed county and tribal boundaries. The "nearest" geographic information systems function was used to find the nearest feature or geometry from one spatial data set to another. It calculates the distance and identifies the closest feature in a reference dataset for each feature in a target dataset. In this example, the "nearest" function was used to determine the distance between each practice location, the closest IHS hospital, and the distance to the nearest subspecialist. The mean driving distances from the 28 IHS hospitals to the nearest OBGYN subspecialist were calculated to evaluate the overall accessibility of subspecialty care for IHS patients. Additionally, the number of OBGYN subspecialists within a driving radius of less than 30 minutes, 30 to 59 minutes, 60 to 89 minutes, 90 to 119 minutes, 120 to 149 minutes, 150 to 179 minutes, 180 to 209 minutes, and 210 to 240 minutes from IHS or tribal hospitals was determined [10].

Driving times and distances

An isochrone is a region of a map that depicts the area accessible by driving the local roads, as opposed to “as the crow flies” from an IHS hospital. Isochrones have been widely used in transport planning since the 1880s to assist with the design of transportation systems [14]. We used isochrones to assist in designing transportation systems and their integration within estimated driving distances from each IHS hospital to the closest OBGYN subspecialist [14]. A 30-minute driving time was chosen as the shortest isochrone, based on the definition of a critical access hospital, typically less than 35 miles away from patients [10]. The upper limit of 240 minutes was based on the average mileage driven with one tank of fuel. Highway driving at 30 miles per gallon in a light-duty truck under good weather conditions was utilized, given the prevalence of this vehicle in the high plains region. Separate analyses were conducted for each IHS hospital, excluding Hawaii and Alaska, due to unique geographies. In Alaska, where ground transportation was limited, fixed-wing aircraft flight routes were substituted. Isochrones, depicting the area accessible by driving local roads, were used to determine estimated driving distances from each Census block group to the closest OBGYN subspecialist. There are no IHS hospitals or reservations located in the state of Hawaii, and therefore, it was excluded. However, it's worth noting that healthcare services for Native Hawaiians are primarily administered through the Department of Hawaiian Home Lands and the Native Hawaiian Healthcare Systems [5].

Mean wait times

A mystery caller study was conducted to determine the mean wait times for new appointments and the types of insurance accepted by the OBGYN subspecialists nearest each IHS facility. While accounting for time zones, phone calls were placed during standard working hours (8 a.m.-5 p.m., except for the 12-1 p.m. lunch hour, local time) from October 11, 2021, to November 15, 2021. We postulated that this one-month calling period ensured that long intervals between calls or seasonal variations in medical care did not influence estimated appointment times. A call was considered unanswered if the caller was on hold for more than five minutes. Each physician's office had two attempts to answer each insurance call, after which the physician was deemed unreachable. No appointments were scheduled to minimize the administrative burden on subspecialty offices, and no patient names or identifying information were provided. One single caller (AA), representing a patient with IHS insurance, requested the soonest available appointment for non-emergent conditions. The caller asked if the subspecialist provided care at the nearby IHS hospital. Ethical safeguards were implemented, including minimizing the time spent on the phone and providing debriefing letters to the offices after the completion of calls. The clinical vignettes for each subspecialty were selected based on standard, non-urgent diagnoses (Appendix).

Statistical analysis

R version 4.2.2 (RStudio, Boston, MA) was used for network analyses, map construction, and all other statistical analyses. REDCap electronic data capture tools were utilized for data collection and management of the mystery caller study component [15]. REDCap is a secure, web-based application that supports data capture in research studies [16].

## Results

**Figure 1:**
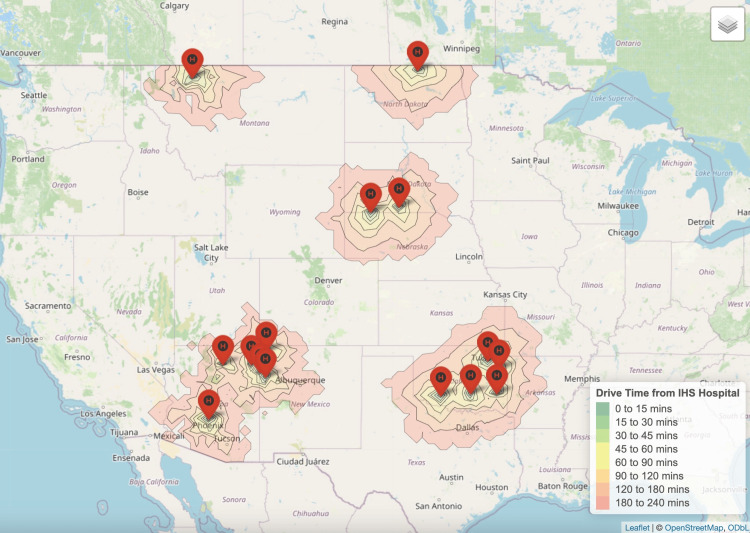
Plot of each Indian Health Service Hospital providing obstetrics and gynecology care.

A total of 37 OBGYN subspecialists located closest to the IHS hospitals providing OBGYN services met the inclusion criteria and were contacted. Among them, subspecialists were closest, with ten in gynecologic oncology, nine in maternal-fetal medicine, eight in urogynecology, five in pediatric and adolescent gynecology, and five in reproductive endocrinology and infertility (Figure 1); pediatric, adolescent, and women.

Based on US Census data, we identified a total of 583,574 pediatric, adolescent, and women from the AI/AN population residing within a 60-minute drive of an IHS hospital. Remarkably, this represents approximately one-quarter of the entire female AI/AN population. To put this into perspective, the number of over half a million women surpasses the combined female populations of Wyoming and Vermont [1]. The median driving time to any of the closest OBGYN subspecialists was 214 minutes (IQR 107-290).

Gynecologic oncology

**Figure 2:**
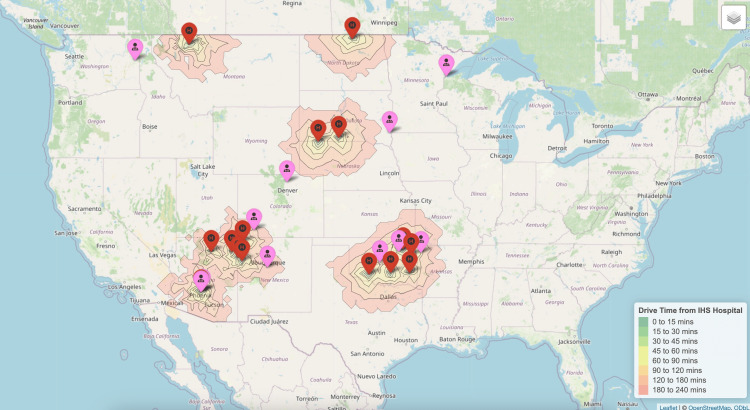
Nearest gynecologic oncologists to each Indian Health Service Hospital.

Around 6% of AI/AN women over 45 who may require gynecologic oncology services resided near IHS hospitals (Figure 2). Nationally, there were approximately 1,053 gynecologic oncologists, resulting in a ratio of 2 gynecologic oncologists per 100,000 AI/AN women over 45. The median driving time to the nearest gynecologic oncologist from an IHS hospital was 206 minutes (IQR 92-328).

Maternal-fetal medicine

**Figure 3:**
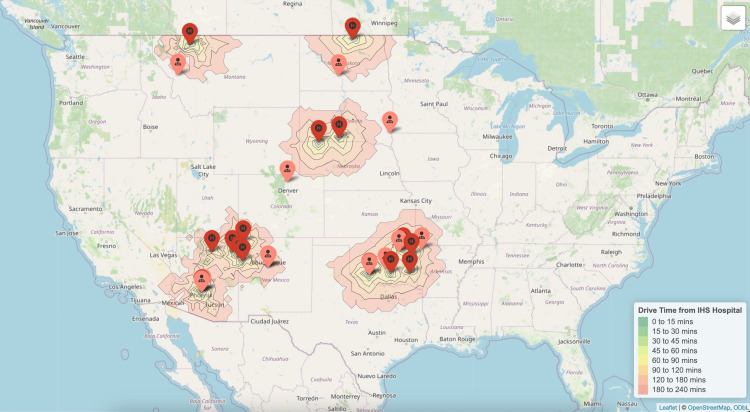
Nearest maternal-fetal medicine subspecialists to each Indian Health Service Hospital.

Maternal-fetal medicine subspecialist providers had a slightly shorter mean driving time of 183 minutes (IQR 56-257) from rural IHS or tribal hospitals than subspecialists (Figure 3).

Pediatric and adolescent gynecology

**Figure 4:**
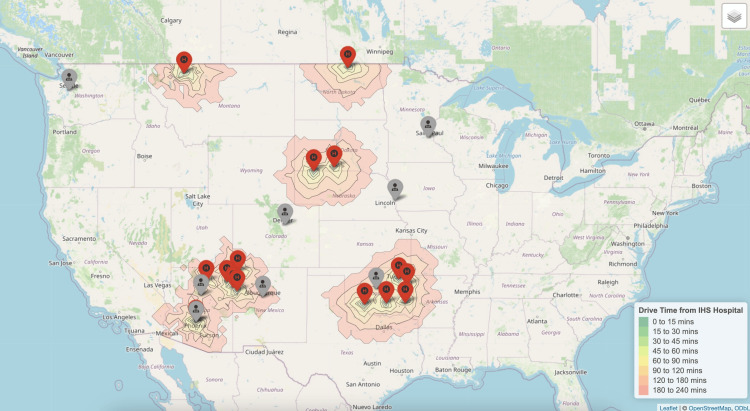
Nearest pediatric and adolescent gynecology subspecialists to each Indian Health Service hospital.

Approximately 200,000 AI/AN pediatric and adolescent individuals under the age of 18 were located within a 60-minute drive of the 17 IHS hospitals providing obstetric and gynecologic services (Figure 4).

This population accounted for 23% of the total AI/AN female population within a 60-minute drive. The median driving time to the nearest pediatric and adolescent gynecologist from rural IHS or tribal hospitals was 216 minutes (IQR 138-394).

Reproductive endocrinology and infertility

**Figure 5:**
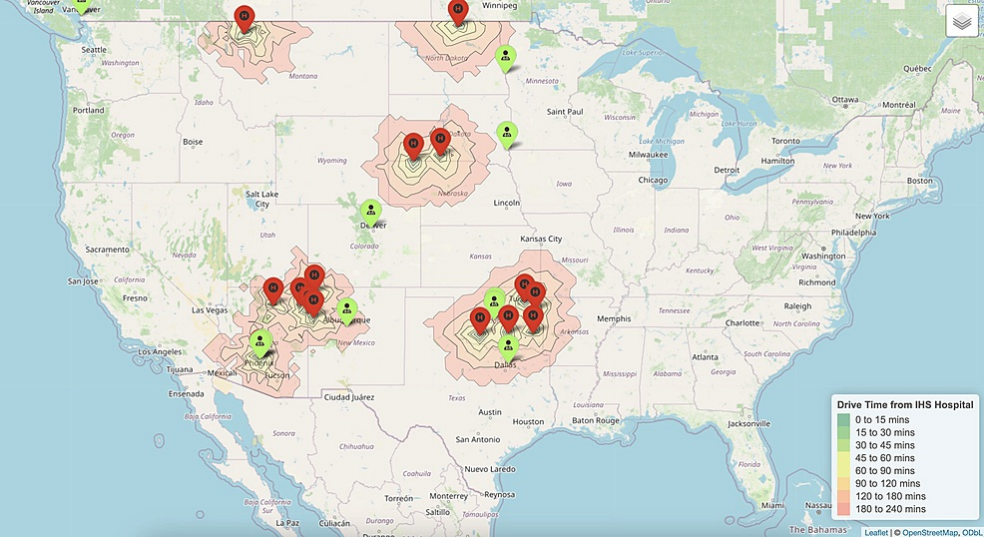
Nearest reproductive endocrinology and infertility subspecialists to each Indian Health Service Hospital.

The median driving time to the nearest REI physician from rural IHS or tribal hospitals was 234 minutes (IQR 143-281) (Figure 5).

Urogynecology

**Figure 6:**
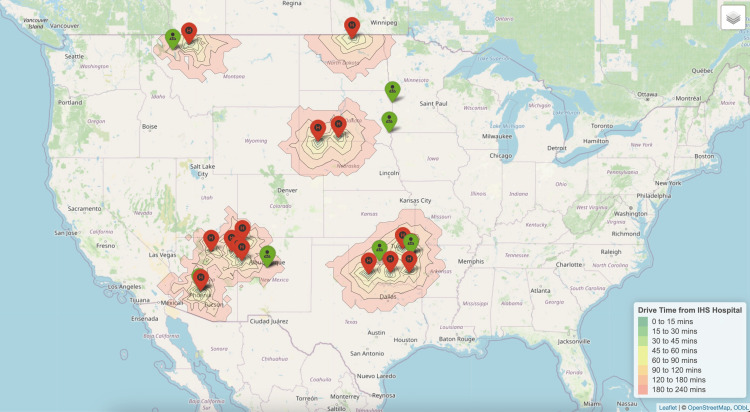
Nearest urogynecology subspecialists to each Indian Health Service Hospital.

Urogynecology had the longest median drive time among the subspecialties, with a value of 915 minutes (IQR 707-1150) (Figure 6). A total female population of 40,631 AI/AN individuals over 45 years old resided within a 60-minute drive of an IHS hospital. Only 5.7% of this population lived within a 240-minute drive of a urogynecology clinic provider. Approximately 76% of IHS or tribal hospitals did not have a urogynecology physician within a 60-minute driving radius, and 35.3% lacked a urogynecology surgeon within a 240-minute driving radius. We identified 51 urogynecologists within a four-hour drive of an IHS hospital, representing approximately 6.9% of the total urogynecology workforce.

Mystery caller results

Of the 37 subspecialists, 89% (n=33) were successfully contacted on the first attempt, and the remaining 11% (n=4) required a second attempt (Table 2).

**Table 2:** Business days until new patient appointment at the nearest obstetrician-gynecologist subspecialists.

Subspecialty	Business days until new patient appointment	
	Mean	Standard deviation
Gynecologic oncology	13	15
Maternal-fetal medicine	13.4	9
Pediatric and adolescent gynecology	18	18
Reproductive endocrinology and infertility	11.5	6
Urogynecology	8.8	4

We were able to contact all subspecialist offices. Two-thirds of the nearest subspecialists provided care at IHS hospitals. It was found that 37% of the nearest subspecialist clinics did not accept referrals from the IHS PRC program, while 16% of the subspecialists did not accept Medicaid insurance. 48% of subspecialty OBGYN clinics accepted self-paying patients. Additionally, 29% of subspecialty providers required a referral or PRC before scheduling an appointment. The mean wait time for a new patient appointment was 13.6 business days (SD ± 2), with no statistically significant difference observed in mean wait time among the subspecialties (p = 0.82).

## Discussion

The results of the mystery caller study revealed that the mean wait time for a new appointment with a subspecialty OBGYN clinic was approximately 14 business days, comparable to the national average of 14 days. The geographic analysis further revealed that only 37 subspecialty clinics were located within a 240-minute drive from the 17 IHS hospitals. The drive time to travel is prohibitive. The distance required to reach these clinics imposes substantial patient costs, including time, money, and potential time away from work or family responsibilities. Additionally, limited access to transportation options and weather-related road conditions can further exacerbate these challenges. It is important to note that while subspecialty care is crucial, non-subspecialists should also be prepared to provide adequate care for these conditions. These findings are consistent with literature identifying distance as a critical barrier to healthcare access for rural populations [17]. Medicare patients in rural areas travel 2-3 times farther to see medical or surgical specialists than their urban counterparts [18]. Most OBGYN subspecialists practice in urban counties [1].

Small differences in distance can affect access to obstetric and gynecologic care [19]. For example, models demonstrate that those who live further from a gynecologic oncologist have a significantly greater risk of death than those who live less than 30 miles from a gynecologic oncologist [20]. Insurance may pose an additional barrier for AI/AN patients who can commute to these distant clinics because of the abovementioned challenges with approval and payment for IHS PRC referrals for specialty care. In this study, 37% of the nearest subspecialist clinics did not accept IHS PRC referrals, and 16% of clinics did not take Medicaid insurance. Medicaid is a vital insurer for AI/AN patients as it enables access to a broader array of services and increases revenues for the IHS and tribal facilities. Less than four of 10 AI/AN patients (36%) have private coverage, compared to 62% of the overall less than 65-year-old population [21]. Medicaid helps fill this gap, covering one in three less than 65-year-old AI/AN patients [21]. Research has shown that access to rural specialty care is limited, with less than one-third of IHS physicians reporting good access to specialty care. Furthermore, over half of these physicians noted that the complexity of care they manage without specialty input is greater than it should be [22]. Lastly, a 2016 US Government Accountability Office report noted that IHS has not conducted any systematic, agency-wide oversight of the timeliness of primary care provided in its federally operated facilities and, as a result, cannot ensure that patients have access to timely primary care.

This study has significant limitations. First, we relied on the self-reported taxonomy codes because no national public database of subspecialty board-certified obstetrician-gynecologists exists. Second, there has likely been an increase in general obstetrician-gynecologists treating patients with these conditions due to more subspecialty residency training. Also, there is likely an increase in the number of AI/AN populations that can be reviewed with the 2020 US Census. We encourage the development of incentives to attract subspecialists to areas with high AI/AN populations, such as offering loan forgiveness programs or financial incentives. These concerns conservatively bias our estimates of driving times and distances to the closest OBGYN subspecialist. Strategies that promote the use of telemedicine for OBGYN subspecialty consultations, especially in remote or underserved areas, may be most beneficial. Third, this study does not evaluate other patient-sided barriers to care, such as language barriers, lack of access to transportation, or mistrust in the healthcare system, given the historical precedence of unethical research and medical practice performed on AI/AN populations. Despite these limitations, the present study has several unique strengths. The strength of this study was the mystery caller format, which closely represented patient experience while making appointments with providers. We estimated travel times using road networks, giving accurate real-life estimates of the distance traveled. Additionally, we used specific geographic locations for subspecialists instead of sites approximated by postal ZIP codes. We also evaluated timely access based on population size rather than land coverage.

This study identified several barriers to OBGYN subspecialty care for AI/AN communities in rural areas, including geographical distance and inconsistent access for those with Medicaid coverage. Focused efforts to address geographic and financial barriers and improve the capacity of frontline OBGYN practitioners may help minimize the disparities in outcomes for obstetric and gynecologic diseases.

## Conclusions

The findings of this study shed light on significant challenges in accessing subspecialty obstetrics and gynecology care for the AI/AN population. The mean wait time for new appointments with subspecialty OBGYN clinics was approximately 14 business days, comparable to the national average of 14 business days. Geographic analysis further revealed that only 37 subspecialty clinics were within a several-hour drive from the 17 IHS hospitals, imposing significant burdens on patients regarding time, money, and potential work or family disruptions. Limited transportation options and weather-related road conditions exacerbated these challenges. Lastly, a 2016 US Government Accountability Office report noted that IHS has not conducted any systematic, agency-wide oversight of the timeliness of primary care provided in its federally operated facilities and, as a result, cannot ensure that patients have access to timely primary care.
